# Identification of seed proteins associated with resistance to pre-harvested aflatoxin contamination in peanut (*Arachis hypogaea L*)

**DOI:** 10.1186/1471-2229-10-267

**Published:** 2010-11-30

**Authors:** Tong Wang, Erhua Zhang, Xiaoping Chen, Ling Li, Xuanqiang Liang

**Affiliations:** 1 Gguangdong Key Lab of Biotechnology for Plant Development, College of Life Science, South China Normal University, Guangzhou 510631, China; 2 Crops Research Institute, Guangdong Academy of Agricultural Sciences, Guangzhou 510640, China

## Abstract

**Background:**

Pre-harvest infection of peanuts by *Aspergillus flavus *and subsequent aflatoxin contamination is one of the food safety factors that most severely impair peanut productivity and human and animal health, especially in arid and semi-arid tropical areas. Some peanut cultivars with natural pre-harvest resistance to aflatoxin contamination have been identified through field screening. However, little is known about the resistance mechanism, which has slowed the incorporation of resistance into cultivars with commercially acceptable genetic background. Therefore, it is necessary to identify resistance-associated proteins, and then to recognize candidate resistance genes potentially underlying the resistance mechanism.

**Results:**

The objective of this study was to identify resistance-associated proteins in response to *A. flavus *infection under drought stress using two-dimensional electrophoresis with mass spectrometry. To identify proteins involved in the resistance to pre-harvest aflatoxin contamination, we compared the differential expression profiles of seed proteins between a resistant cultivar (YJ-1) and a susceptible cultivar (Yueyou 7) under well-watered condition, drought stress, and *A. flavus *infection with drought stress. A total of 29 spots showed differential expression between resistant and susceptible cultivars in response to *A. flavus *attack under drought stress. Among these spots, 12 protein spots that consistently exhibited an altered expression were screened by Image Master 5.0 software and successfully identified by MALDI-TOF MS. Five protein spots, including Oso7g0179400, PII protein, CDK1, Oxalate oxidase, SAP domain-containing protein, were uniquely expressed in the resistant cultivar. Six protein spots including low molecular weight heat shock protein precursor, RIO kinase, L-ascorbate peroxidase, iso-Ara h3, 50 S ribosomal protein L22 and putative 30 S ribosomal S9 were significantly up-regulated in the resistant cultivar challenged by *A. flavus *under drought stress. A significant decrease or down regulation of trypsin inhibitor caused by *A. flavus *in the resistant cultivar was also observed. In addition, variations in protein expression patterns for resistant and susceptible cultivars were further validated by real time RT-PCR analysis.

**Conclusion:**

In summary, this study provides new insights into understanding of the molecular mechanism of resistance to pre-harvest aflatoxin contamination in peanut, and will help to develop peanut varieties with resistance to pre-harvested aflatoxin contamination.

## Background

Peanut (*Arachis hypogaea *L.) is one of most important and widespread oil crops. One of the major problems in peanut production worldwide is aflatoxin contamination, which is of great concern in peanut as this toxin can cause teratogenic and carcinogenic effects in animal and human. Infection of peanut by *Aspergillus flavus *occurs not only in post-harvest but also in pre-harvest conditions [1-3]. Several biotic (soil-born insects) and abiotic (drought and high temperature) factors are known to affect pre-harvest aflatoxin contamination, while the late season drought (20-40 days before harvest) which predispose peanut to aflatoxin contamination [4-9] is more important in the semi-arid tropics [10,11]. Irrigation in late season can reduce peanut pre-harvest aflatoxin contamination, but this cultural practice seems to be impractical in some areas, especially in semi-arid and arid areas. Enhancing host plant resistance to pre-harvest *A. flavus *invasion and aflatoxin contamination is considered to be the most cost-effective control measure. In the past decades, peanut cultivars with natural pre-harvest resistance to aflatoxin production have been identified through field screening [12-21]. However, the agronomic traits of these varieties have been very poor for the direct commercial utility. The progress in transferring the resistance genes from these resistant lines into commercial cultivars has been slow, due to lack of understanding of the resistance mechanism and markers associated with resistance [22].

Although drought stress is known to predispose peanut to aflatoxin contamination [4-9], limited researches were reported on the mechanism of late season drought stress aggravating the *A. flavus *infection. Dorner *et al *(1989) [23] observed that drought stress could decrease the capacity of peanut seeds to produce phytoalexins, and thus resulted in higher aflatoxin contamination. The active water of seeds is the most important factor controlling the capacity of seeds to produce phytoalexins [23,24]. Luo *et al *(2005) [25] used a microarray of 400 unigenes to investigate the up/down regulated gene profiles in peanut cultivar A13, which is drought tolerant and resistant to pre-harvest aflatoxin contamination, and identified 25 unigenes that were potentially associated with drought tolerance or that responded to *A. parasiticus *challenge. Nevertheless, the significance of these unigenes in pre-harvest infection of peanut pods by *Aspergillus *is incomplete without knowledge of their functions. Studies to understand host resistance mechanisms in maize and peanut against *A. flavus *infection and aflatoxin contamination indicate that proteins are a major factor contributing to kernel resistance [1,2,26,27].

Proteins serve as the bridge between genetic information encoded in the genome and the phenotype. Proteomics analysis reveals the plasticity of gene expression as it allows global analysis of gene products and physiological states of plant under particular conditions. The objectives of this research were to: (1) compare the differential expression of proteins of resistant and susceptible peanut cultivars in response to *A. flavus *challenge under drought stress; (2) identify seed proteins associated with resistance to pre-harvest aflatoxin contamination in peanut. In this study, a total of 28 differentially expressed proteins were identified and 12 proteins associated with pre-harvested aflatoxin contamination were further characterized by MALDI-TOF MS and their expression profiles were validated by real-time RT-PCR. The identification of these potential proteins associated with the aflatoxin resistance in peanut could be useful in programmes on developing peanut varieties with resistant to pre-harvest aflatoxin contamination.

## Results

### Aflatoxin accumulation analysis in seeds of resistant and susceptible cultivars

Seed aflatoxin B1 levels from the resistant cultivar (YJ-1) and susceptible cultivars (Yueyou 7) had baseline levels (approximately 1 ppb) under well-watered conditions, and no difference between the two cultivars was found (Table 1). Under drought stress conditions, the seed aflatoxin B1 level in both YJ-1 and Yueyou 7 increased. The level of aflatoxin B1 increased to 22 ppb and 162 ppb in YJ-1 and Yueyou 7 respectively under drought stress. After artificial inoculation treatment with *A. flavus *under drought stress, the aflatoxin B1 level in seeds of the infected cultivar YJ-1 increased to 135 ppb, whereas the level in the infected cultivar Yueyou 7 increased to 1901 ppb, suggesting that aflatoxin B1 accumulation in the susceptible cultivar Yueyou 7 was around 14-fold compared to the resistant cultivar YJ-1. YJ-1 exhibited a significant level of resistance to pre-harvest aflatoxin contamination. These results are in agreement with several earlier reports of resistance in peanut [28].

**Table 1:** Mean aflatoxin B1 contamination of resistant and susceptible cultivars planted at different condition in 2008/2009 season at Guangzhou, China.

Treatments	Mean aflatoxin B1 contamination (ppb)	
	
	Resistant cultivar YJ-1	Susceptible cultivar Yueyou7
Well-watered condition	1.2	1.3
Drought-stress	22	162
*A. flavus *inoculation under drought stress	135	1901

### Comparison of seed proteomic profiles between resistant and susceptible cultivars under *A. flavus *challenge and drought stress

**Figure 1:**
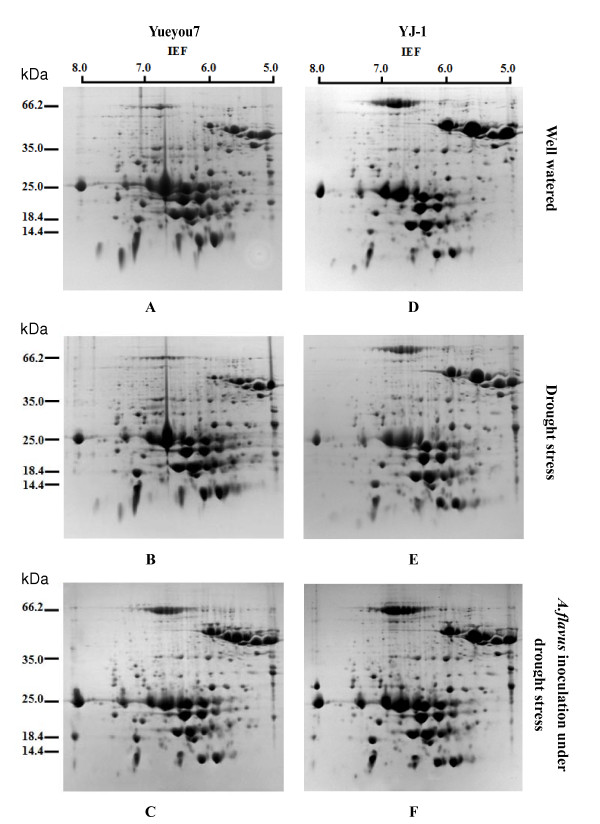
**2-DE analysis of peanut seed proteins from the susceptible cultivar YueyouY7 (a, b and c) and the resistant cultivar YJ-1 (d, e and f) challenged with *A. flavus *and drought stress(c, f), drought stress alone (b, e) and untreated as control (a, d)**. Proteins were separated in the first dimension on an IPG strip pH 5-8 and in the second dimension on a 15% acrylamide SDS-gel, followed by staining with Coomassie brilliant blue G-250 stain. An equal amount (200 ug) of total protein extracts was loaded in each gel. The gels were scanned and the images were analyzed using Image Master 2 D Platinum 5.0 software.

To investigate the seed protein profiles, we carried out 2-DE analysis of the proteins from six sample groups as described in the Methods section. Due to the lower resolution at the anodal and cathodal ends of the first dimension tube gels, only the gel region where the pI ranged from 5 to 8 was further analyzed. For each treatment, 2-DE gels were run in three replicates. More than 500 protein spots were repeatedly detected on Coomassie brilliant blue G-250 -stained gels using Image Master 5.0 software across all the samples (Figure 1) and the reproducibility of all gels were over 95.0% (Additional file 1).

A comparison of 2-DE images revealed that there were both qualitative and quantitative differences in resistant or susceptible cultivars under the three treatment conditions (Additional file 2). Under the well-watered condition, the 2-DE gel of resistant cultivar YJ-1 showed 542 high quality spots (Additional file 1), while 11 unique, 12 up-regulated, 6 down-regulated and 6 disappeared spots were induced by drought stress, 17 unique, 15 up-regulated, 5 down- regulated and 7 disappeared spots were induced by *A. flavus *infection under drought stress (Additional file 2). The 2-DE protein profiles of the susceptible cultivar (Yueyou 7) showed a similar differential expression pattern responsive to drought stress and *A. flavus *infection, but the number of differentially expressed spots was less than that of the resistant cultivar (YJ-1). Five unique, 10 up-regulated, 5 down-regulated and 3 disappeared spots were induced by drought stress, while 12 unique, 11 up-regulated, 8 down-regulated and 4 disappeared spots were induced by *A. flavus *infection under drought stress in susceptible cultivar Yueyou 7 (Additional file 2).

**Figure 2:**
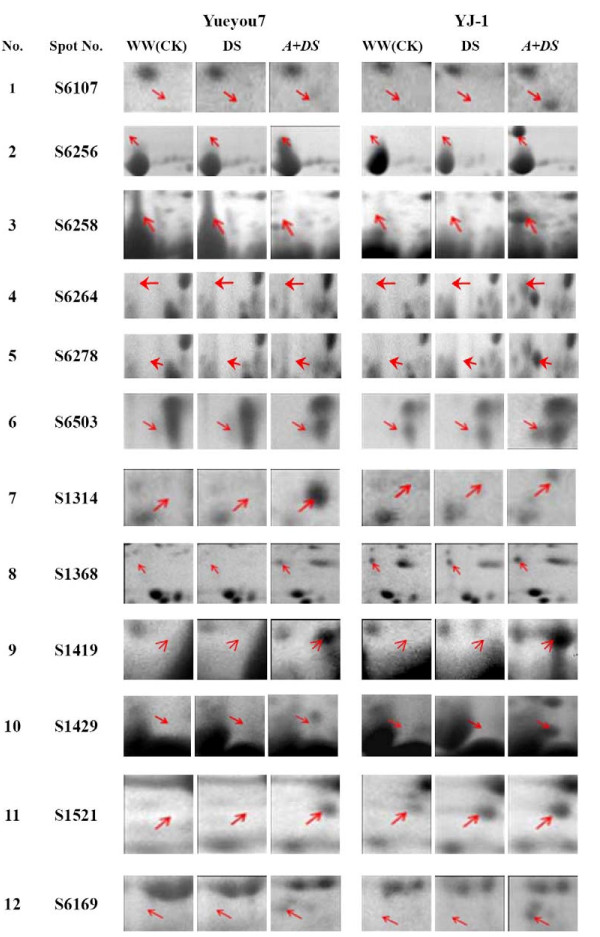
**The enlargements of twelve differentially expressed proteins spots in response to *A. flavus *invasion under drought stress condition**. The arrows indicate the proteins that were differentially expressed. WW (CK): well-watered condition (control); DS: drought stress; *A*+DS: drought stress and *Aspergillus flavus *infection. Yueyou7: susceptible cultivar; YJ-1: resistant cultivar.

To investigate the host proteins responsive to *A. flavus *infection, a comparison was conducted with 2-DE images of total seed proteins from the resistant cultivar (YJ-1) and the susceptible cultivar (Yueyou 7) with *A. flavus *infection under drought stress (Table 2). About 29 spots that showed differential expression in all analytical gels under *A. flavus *attack were identified. Among those, 12 protein spots that consistently exhibited unique, increased or decreased in abundance and at least four fold differences in spot intensity in gel of resistant cultivar (YJ-1) with *A. flavus *infection under drought stress, compared with gel of the susceptible cultivar (YY-7) received the same treatment. Of these, five protein spots (S6256, S6258, S6264, S6278, and S6503) with unique expression, six protein spots (S1368, S1521, S1419, S1429, S16169 and S6107) with an up-regulated trend, and one protein spots (S1314) with a down-regulated trend in the resistant cultivar (YJ-1) by *A. flavus *infection under drought stress were selected for MS analysis. The enlargements of the 12 differentially expressed proteins were shown in Figure 2.

**Table 2:** Differential expression spots of resistant cultivar YJ-1 compared to susceptible cultivar Yueyyou7 in response to A. flavus invasion under drought stress condition

	Differential expression spots in YJ-1 compared to Yueyou 7	Selected for MS analysis
No. of unique express spot	8	6
No. of up regulated spot	10	5
No. of down regulated spot	7	1
No. of miss spot	4	
Total	29	12

### Identification of the differentially expressed proteins related to resistance to pre-harvest aflatoxin contamination

All of the twelve differentially expressed proteins were excised and analyzed by MALDI-TOF-MS to identify their putative functions. After searching against the green plant protein database in NCBI, all these protein spots were successfully identified by PMF analysis and matched known plant proteins. Those proteins and their annotated functions are listed in Table 3. Since there are relatively few known peanut proteins and genomic sequences available, only three proteins matched peanut proteins. Among the twelve selected proteins, four were related to stress response: Low molecular weight heat shock protein precursor (S6107), Oxalate oxidase (S6278), Trypsin inhibitor (S1314) and L-ascorbate peroxidase 1(S1521). Os07g0179400 (S6256), CDKD1 (S6264) and RIO kinase (S1368) were signaling components. SAP domain-containing protein (S6503), 50 S ribosomal protein L22 (S1429) and putative 30 S ribosomal protein S9 (S6169) were related to regulation of transcription. PII protein (S6258) and iso-Ara h3 (S1419) were storage protein.

**Table 3:** Differentially expressed proteins of peanut seed under infection by A. flavus identified by MALDI-TOF MS*.

**No**. ^**a**^	**Accession No**.	Homologous protein	Organism	Description of potential function	**Theo. Mr (kD)/pI**^**b**^	**PM**^**c**^	**SC(%)**^**d**^	Protein Score
S6107	AAC12279.1	Low molecular weight heat shock protein precursor	*Zea mays*	Stress response	23.8/6.5	10	37.1	55
S6256	NP_001059035.1	Os07g0179400	*Oryza sativa*	Signaling components	20.0/5.1	9	36.36	58
S6258	AAC78332.1	PII protein	*Arabidopsis thaliana*	Unclassified	21.7/8.9	10	38.1	60
S6264	NP_177510.1	CDKD1	*Arabidopsis thaliana*	Signaling components	45.1/9.4	16	27.1	76
S6278	ABS86850.1	Oxalate oxidase	*Arachis hypogaea*	Defense response	23.1/7.7	14	23	80
S6503	NP_201151.2	SAP domain-containing protein	*Arabidopsis thaliana*	Regulation of transcription	17.5/9.8	12	39.5	70
S1314	AAM93157.1	Trypsin inhibitor	*Arachis hypogaea*	Defense response	25.5/6.7	10	37.9	81
S1368	BAD12556.1	RIO kinase	*Nicotiana tabacum*	Signaling components	66.6/5.5	18	23.3	66
S1419	ABI17154.1	Iso-Ara h3	*Arachis hypogaea*	Unclassified, storage protein	58.2/5.4	10	24.8	96
S1429	P49163	50 S ribosomal protein L22	*Medicago sativa*	Regulation of transcription	21.8/10.3	12	27.5	73
S1521	Q05431	L-ascorbate peroxidase 1	*Arabidopsis thaliana*	Defense response	27.5/5.7	10	25.6	56
S6169	BAC81159.1	Putative 30 S ribosomal protein S9	*Oryza sativa*	Regulation of transcription	45.0/5.5	16	25.5	71

a: Spot number; b: Theoretical molecular weight/isoelectric point; c: Number of matched peptides; d: Sequence coverage.

### Gene Transcription Profile Analysis by real time RT-PCR

**Figure 3:**
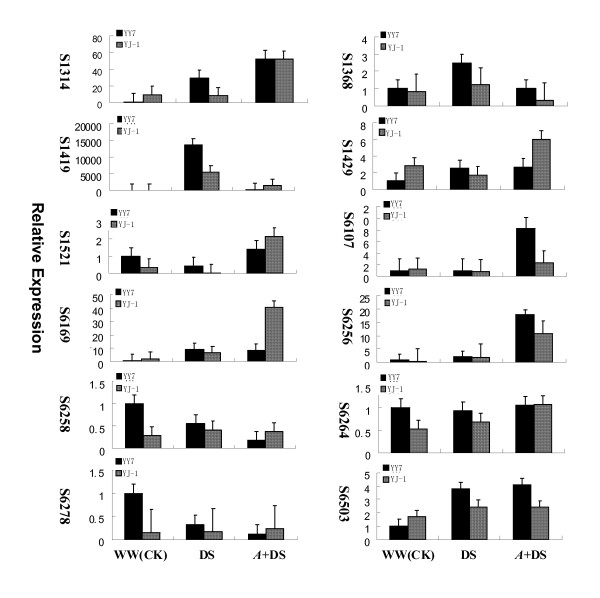
**Real time RT-PCR analysis on mRNA transcription of the differentially expressed proteins in response to *A. flavus *invasion under drought stress condition**. Total RNA were isolated from the seeds of resistant (YJ-1) and susceptible (Yueyou7, YY7) cultivar at 50 days post-treatments. Twelve genes were selected for real time RT-PCR analysis to study the relationship between protein expression and gene transcription and the expression levels normalized using *actin *gene as the internal control. The expression of these genes in Yueyou 7 under well-watered conditions was used as the target calibrator. Real-time PCR analyses were performed based on three replicates.

To validate the expression of the twelve identified proteins at transcription level, total RNAs from six samples (see the Methods section) were extracted and analyzed by real time RT-PCR. The primer pairs used for real time RT-PCR were designed based on nucleotide sequences in NCBI databases and shown in Table 3 the *actin *gene was chosen as internal control. Figure 3 shows the expression patterns of the twelve genes in the resistant cultivar (YJ-1) and the susceptible cultivar (Yueyou7) under well-watered (control), drought stress and *A. flavus *infection accompanied with drought stress on the 50^th ^days after treatments. The results demonstrated that, of the five genes identified as the unique expressed group (S6256, S6258, S6264, S6278, and S6503), S6258 and S6278 showed higher expression levels in the cv. YJ-1 than in the cv. Yueyou7, S6264 showed similar and the remaining two showed lower. Of the six proteins identified as the up-regulated group (S1368, S1521, S1419, S1429, S6107 and S6169), four genes (S1521, S1419, S1429, S6169) showed higher expression levels in the resistant cultivar with *A. flavus *infection under drought stress. In contrast, two genes (S1368 and S6107) showed no correlation between mRNA and protein expression levels. One gene (S1314) identified in the down-regulated group, showed the identical level of transcript abundance in both resistant and susceptible cultivars with *A. flavus *infection plus drought stress.

## Discussion

In this study, proteins showing differentially expressed profiles in the resistant and susceptible cultivars with *A. flavus *infection under drought stress were identified by using a proteomic approach. Around 550 protein spots identified for quantitative analyses of differentially regulated proteins responsive to *A. falvus *attack, and the number of protein spots was more than that in earlier reports by Liang *et al *(2006b) [29] and Kottapalli *et al *(2008) [30]. We have identified 12 protein spots which significantly increased or decreased in response to *A. flavus *infection under drought stress in resistant cultivar (YJ-1) versus susceptible cultivar. These proteins could be divided into four functional groups including defense response, signaling components, regulation of transcription and storage protein.

Os07g0179400 (s6256) with transferase and kinase activity is a key protein in biosynthetic process [31]. CDKD1 (s6264) is involved in the phosphorylation of proteins and regulation of cell cycle [32]. Oxalate oxidase (s6278) belongs to the germin-like family of proteins and catalyzes the degradation of oxalic acid to produce carbon dioxide and hydrogen peroxide [33]. Reports of oxalate oxidase activity in response to pathogen attack have received considerable attention as it possibly plays a role in plant defense [34-37]. In plants, PII protein (s6258) is a nuclear-encoded plastid protein [38] and can be involved in the regulation of nitrogen metabolism [39]. SAP domain-containing protein (s6503) was a DNA binding protein and its physiological roles remain to be unknown. In this study, these five proteins had unique expression in resistant cultivars and completely absent in the susceptible cultivar in response to *A. flavus *infection under drought stress, or under only drought stress condition. These proteins were, therefore, considered to be encoded by candidate resistance-related genes potentially involved in resistance to preharvest aflatoxin contamination.

Heat shock proteins (s6107), 50 s ribosomal protein (s1429), 30 s ribosomal protein (s6169) and iso-ara h3 (s1419) were up-regulated in both cultivars only in *A. flavus *infection under drought stress condition, but the expression level in the resistant cultivar was higher than in susceptible cultivar. Heat shock proteins (HSP) are the most well-known stress related proteins in plants which are induced in response to a number of different stresses. HSP can play a role as chaperons which are involved in correct folding of proteins and protect them from denaturing under stress condition [40]. In this study, HSP proteins could only be observed in peanut seeds upon *A. flavus *attack under drought conditions. This result was contradictive with those of Chen *et al *(2002, 2007) [41,26], in which they reported that HSP proteins were constitutively expressed and up-regulated in resistant maize lines versus susceptible lines [26,41]. Both 50 S ribosomal protein (s1429) and putative 30 S ribosomal protein (s6169) are structural constituents of ribosome with RNA binding function, and play essential roles in translation processes [42]. The transcripts of ribosomal proteins in leaves of *Arabidopsis *plants were up-regulated under both drought and heat stress conditions [43]. The significant up-regulation of two ribosomal proteins suggested that one of the major effects of pre-harvest *A. flavus *infection in peanut is imposed on protein synthesis. Iso-Ara h3 (s1419), a peanut seed storage protein, shows significant homology to known peanut allergen, Arah3 [29]. The significant increase of iso-ara h3 in resistant cultivar compared with susceptible cultivar under *A. flavus *infection showed that iso-ara 3 (s1419) might be related to pre-harvest aflatoxin contamination.

L-ascorbate peroxidase (s1521) is a stress-responsive protein [44], and is involved in the metabolism of H_2_O_2 _in higher plants [45]. Previous reports on peanut [24] and maize [26] showed L-ascorbate peroxidase were up-regulated by both *A. parasticus *and drought stress. RIO kinase (s1368) has kinase catalytic activity and is involved in ATP binding [46,47]. In this study, L-ascorbate peroxidase (s1521) and RIO kinase (s1368) were detected only in the resistant cultivar under well-watered conditions, and were up-regulated under drought stress conditions and *A. flavus *attack under drought stress conditions. In the susceptible cultivar, however, the two proteins were up-regulated only under *A. flavus *attack accompanied with drought stress. This result was consistent with previous studies [24,26]. This indicated that the two proteins (s1521 and s1368) might contribute to increasing the resistance to pre-harvest aflatoxin contamination in the resistant cultivar.

Trypsin inhibitor (s314), a constitutively expressed antifungal protein, was observed at high expression levels in resistant peanut cultivars [48] and maize lines [49,41], but was at low or undetectable levels in susceptible cultivars and lines. However, in this study, there was no differential expression in both cultivars under well-watered and drought stress conditions, but down-regulation of trypsin inhibitor was observed when challenged by *A. flavus *under drought stress in resistant cultivar. The true reason of down-regulation of trypsin inhibitor in our experiment remains unknown.

The functional distribution of unique and up-regulated proteins in resistant cultivar (YJ-1) also showed that most of the proteins affected were defense-related proteins, protein synthesis, and regulation of transcription. *A. flavus *infection in pre-harvested peanut seeds resulted in expression of six new proteins, no information of which was available in database. Three of them (spot s6256, s6258 and s6264) were detectable only in resistant cultivar, and three proteins (s1368, s1429 and s6169) were markedly up-regulated in resistant cultivar.

In addition, in this study, seven selected proteins for mRNA expression study showed up-regulation in both mRNA and protein expression, although it has been reported that the correlation between transcription and translation is known to be less than 50% [50].

## Conclusion

In conclusion, pre-harvest aflatoxin-resistance trait was characterized as a quantitative trait. Development of peanut cultivars with resistance to pre-harvest aflatoxin contamination would be a long-term selection program. This study reports the first proteome analysis to identify resistance-associated protein such as low molecular weight heat shock protein, Oso7g0179400, PII protein, CDK1, Oxalate oxidase, SAP domain-containing protein, RIO kinase, L-ascorbate peroxidase, iso-Ara h3, 50 S ribosomal protein, 30 S ribosomal, which may be associated with resistance to pre-harvest aflatoxin contamination in peanut. More detailed analysis of the identified proteins is in progress to further characterize their possible functional roles in resistance to pre-harvested aflatoxin contamination.

## Methods

### Plants material and treatment

A resistant cultivar YJ-1 and a susceptible cultivar YueyouY-7 were provided by Crops Research Institute, Guangdong Academy of Agricultural Sciences (GDAAS, China). *A. flavus *isolate As3.2890, a wild-type strain known to produce high levels of aflatoxin in peanut was provided by Institute of Microbiology, Chinese Academy of Sciences. All seeds were sterilized for 1 min in 70% ethanol, rinsed with sterile deionized water 3- 4 times. Seeds were planted in plastic pots with sterilized soil and kept in the greenhouse at a temperature of 25-30°C. Both resistant (YJ-1) and susceptible (Yueyou 7) cultivars were subjected to three treatments: (1) well-watered condition; (2) drought stress condition; (3) drought stress and *A. flavus *artificial inoculation condition. To simulate the late season drought, we watered the spots of the drought treatments with only 20 ml of water per day starting on the 60^th ^day after sowing, while the spots of the well-watered treatments were watered normally. In *A. flavus *inoculation group, both cultivars were subjected to drought stress as group 2. In addition, *A. flavus *(As3.2890)-contaminated corn powder was sprayed to pots at 60 days after planting and covered with soil according to the method of Anderson *et al *(1996) [51]. All treatments were conducted simultaneously. The mature seeds were collected and immediately frozen in liquid nitrogen, and then stored in a freezer at -80°C.

### Measurement of aflatoxin B1

Peanut seeds (5 g) of all samples were sprayed with 95% alcohol and dried at 115°C. The dried seeds were ground to powder, defatted with 20 ml of n-hexane, and then extracted with 25 ml of aqueous methanol (1:1). Aflatoxin B_1 _(AFB_1_) extracts of all the samples were determined according to the manufacturer's directions of Aflatoxin B_1 _quantization ELISA Kit (JSWSW, Jiangsu China).

### Seed total protein extraction

The frozen peanut seeds (1 g) of all samples were homogenized in a chilled mortar and ground to powder in liquid nitrogen and defatted with hexane according to Liang *et al *(2006b) [29]. The defatted samples were collected by centrifugation (10,000 × g for 10 min at 4°C and the pellets were allowed to dry at room temperature. The dried pellets were further ground with pestle to a fine powder and re-suspended in 2 ml of phenol for extraction of proteins based on a method modified from Sonia *et al *[52]. The supernatant was collected after centrifugation at 10,000 × g for 10 min at 4°C and precipitated with five volumes of ice-cold methanol plus 0.1 M ammonium acetate at -20°C for 1 h. Precipitated proteins were recovered by centrifugation at 10,000 × g for 10 min at 4°C, and then washed five times with cold methanol, cold acetone and cold 80% acetone. The pellets were vacuum-dried and re-dissolved in 6 M guanidinium chloride. Then 5 mM TBP and 100 mM 2-VP (SIGMA, USA) were added to reduce and alkylate proteins and, after incubating for 90 min at room temperature, supernatant was collected by centrifuging at 10,000 × g for 10 min at 4°C. The supernatant was mixed with five volumes of ice-cold acetone: ethanol (1:1) to precipitate proteins at -20°C for 10 min. The precipitated proteins were recovered and washed twice with cold acetone/ethanol (1:1) and 80% acetone. The final pellets were air-dried and re-suspended in ProteomIQ™C7 re-suspension reagent (Proteome Systems, Inc., Australia) with a drop of ProteomIQ IEF tracking dye. These samples were used for 2-DE analysis.

### Two-dimensional gel electrophoresis (2-DE) and spot analysis

The first-dimensional gel electrophoresis was performed using immobilized pH gradients (Proteome Systems Ltd, Sydney, Australia) according to the manufacturer's directions with some modifications. The dry 11 cm IPG strips (pH5-8) (Proteome Systems Ltd) were rehydrated for 12 h with 200 μl of protein sample, containing 0.3 mg of protein, at 14°C. Isoelectric focusing (IEF) was performed at 20°C with PSL IsoElectrIQ™electrophoresis equipment (Australian). The running conditions were: 1 h at 100 V, 8 h from 100 V to 10,000 V and 8 h at 10,000 V. Current was limited 50 μA per IPG gel strip. The focused strips were equilibrated immediately for 15 min in 10 ml of sodium dodecyl sulfate (SDS) equilibration solution containing 50 mM Tris-HCI buffers, pH8.8, 6 M urea, 2% (wt/vol) SDS, 30% (wt/vol) glycerol, 1% (wt/vol) DTT and a drop of tracking dye at room temperature with shaking.

After equilibration, the second-dimension gel electrophoresis was carried out on 15% polyacrylamide-SDS gels (20 cm × 24 cm × 0.1 cm, width × length × thickness) at a constant voltage of 120 V for 5 h at 20°C.

Preparative gels were fixed overnight in water containing 10% (vol/vol) acetic acid, 50% (vol/vol) methanol, and stained with colloidal Coomassie Brilliant Blue G-250. All the stained gels were scanned and images were analyzed using Image Master 2 D Platinum 5.0 software (Amersham Biosciences). For each sample, gels were run in triplicate.

A comparison of the *A. flavus*-inducing variations between YJ-1 and Yuyou7 allowed the identifation of the induced protein spots that were present uniquely or at least four-fold up/down-regulated in the resistant cultivar compared to susceptible cultivar. For comparison of gels, the intensity data of individual protein spots present in each gel were normalized according to Image Master Software user manual. Intensity of all protein spots were interpreted by a percentage. Then the percent intensity volume (% vol) of each individual spot (relative to the intensity volumes of all spots) was used for the comparative analysis with unpaired Student's t-test. P values less than 0.05 were considered statistically significant.

### MALDI-TOF MS analysis and protein identification

The unique, down- or up-regulated protein spots in response to *A. flavus *infection in the resistant cultivar were cut and in-gel proteolysed with trypsin. The resulting peptides were analyzed by matrix-assisted laser desorption/ionization-time of flight mass spectrometry (MALDI-TOF MS) (WATERS Corporation, USA) at the Beijing Proteomics Research Center (BPRC, China). The list of peptide masses were transferred into the peptide mass fingerprint search program Mascot http://www.matrixscience.com as data file, and were compared with simulated proteolysis and fragmentation of known proteins in the NCBI-nr database. Search parameters in the program allowed for oxidation of methionine, carbamido-methylation of cysteine, one missed trypsin cleavage, and 0.2 Da of mass accuracy for each peptide mass was allowed. Proteins with a MASCOT high score (> 60) were considered to be the target proteins. Proteins that were matched with a lower MASCOT score were considered tentative. In addition, the identified peptides were used for similarity searches against peanut gene indices generated in our laboratory using tBLASTn algorithm.

### Real Time RT-PCR analysis

Total RNA was isolated from peanut seeds using Trizol reagent (Invitrogen, Carlsbad, CA), and genomic DNA was removed by adding RNase-free DNase I (Takara). And then, the RNA samples were purified with the RNeasy Cleanup Kit (Qiagen). Nano drop ND-1000 Spectrophotometer and agarose gel electrophoresis was performed to test RNA quality as described by Aranda, *et al *(2009) [53]. For all the samples, 4 μg of total RNA was converted to cDNA using PrimeScript II 1^st ^Strand cDNA Synthesis kit (Takara) according to the manufacturer's protocols. Quantitative real-time RT-PCR was performed with SYBR^R ^Premix Ex Taq™II kit (Takara) and a LightCycler 480 instrument (Roche) equipped with Light- Cycler Software Version 1.5 (Roche) based on the manufacturer's instructions [54]. Amplifications reactions were carried out in a total volume of 20 μl. PCR cycling was: 95°C for 10 s, followed by 45 cycles of 95°C for 10 s, 60°C for 10 s, and 72°C for 20 s. Data collection was performed during the annealing phase of the each amplification. Then processing of the melting curve was from 62 to 95°C with reading the intensity of fluorescence every 0.2. All protein-specific primers were designed using the Primer Version 5.0 (PREMIER Biosoft Intern ational) and listed in Table 4. The *actin *gene from peanut seed was used as an internal control for calculating relative transcript abundance. The amplicon of this gene is 104 bp and the primers are: forward (5'-GTTCC ACTAT GTTCC CAGGC A-3') and reverse (5'-CTTCC TCTCT GGTGG TGCTA CA-3'). All real-time PCR reactions were technically repeated three times. The relative quantification of RNA expression was calibrated using formula 2^-ΔΔCt ^method [55].

**Table 4:** Primers used for real time RT-PCR of differentially expressed peanut seed proteins in different treatments

**Spot NO**.	Protein description	forward primer(5'-3')	reverse primer(5'-3')	Length (bp)
S6107	Low molecular weight HSP precursor	GCTGGACTTCGTCGTGGTTG	TGGTCAGGGTGTTCTGCTCC	121
S6256	Os07g0179400	CCGCTCAAGATGATCCCATG	ACTGTGCTGAAGCGGTGAGG	129
S6258	PII protein	ATCGGAACGTGGTTCTCACG	GCCTAAGAATGGCTTCCGCT	132
S6264	CDKD1	GTGCTTCAGCGATTCAACGA	GAGGGATCCGGGTCTGTCAT	131
S6278	Oxalate Oxidase	GTTCCATTGTAACAGGAGCCA	TGAGTCCACCTGGGGCATA	123
S6503	SAP domain-containing protein	CACCAGAGGGCCAGCATATT	GATCCCTCGGTTCCATCCTT	115
S1314	Trypsin inhibitor	AAAATGCGTGCCAGTTCCAG	GGAGGACTAAGCGCGAGAGG	141
S1368	RIO kinase	TGGCTTGACTCCAAGGACGA	GAGAGAGGCTGGAGGGTGGA	125
S1419	Iso-Ara h3	TCCAATGCTCCCCTCGAGAT	TGGGTCGTCCTGCCCTACTT	159
S1429	50 S ribosomal protein L22	TCTCTCTCAATTCTCGCCGC	CACGAATGTGGTGCGTGAAC	117
S1521	L-ascorbate peroxidase 1	TGGCCGGTGTAGTTGCTGTT	CCCATAGCCTTGCCAAACAC	154
S6169	Putative 30 S ribosomal protein S9	AGGAGGCGGTGTTTCAGGTC	TGTCAGGAAGCCAGCGTTTC	112

## Authors' contributions

All authors read and approved the final manuscript. TW participated in conceiving the study, material preparation, sequence analysis and drafting the manuscript. EZ carried out the 2-D analysis. XC participated in conceiving the study, designing the real time PCR primers and data analysis. LL participated in conceiving the study and material preparation. XL participated in conceiving the study, data analysis and drafting the manuscript

## Supplementary Material

Additional file 1 **Reproducibility of two-dimensional gels**. Click here for file

Additional file 2 **Summary of differential expression of proteins in Yueyou7 and YJ-1 in three treatments**. Click here for file
