# Supplementary material for: Identification of seed proteins associated with resistance to pre-harvested aflatoxin contamination in peanut (Arachis hypogaea L)
Source: BMC Plant Biol. 2010 Nov 30;10:267. doi: 10.1186/1471-2229-10-267 (PMC3095339; doi:10.1186/1471-2229-10-267)
Supplement: Additional file 1 — Reproducibility of two-dimensional gels. [file 1471-2229-10-267-S1.DOC]

| Cultivars | Treatment | Average no. of spots* | High quality spots** | Reproducibility (%) |
| --- | --- | --- | --- | --- |
| YJ-1 | Well-watered condition | 560 | 542 | 96.79% |
|  | Drought-stress | 547 | 531 | 97.07% |
|  | *A. flavus* inoculation under drought stress | 558 | 542 | 97.13% |
|  |  |  |  |  |
| Yueyou 7 | Well-watered condition | 551 | 536 | 97.28% |
|  | Drought-stress | 557 | 533 | 95.69% |
|  | *A. flavus* inoculation under drought stress | 532 | 515 | 96.80% |
|  |  |  |  |  |
| Total |  | 3305 | 3199 |  |

*Average no. of spots: average number of spots in three replicate gels of each treatment.

** High quality spots: spots having a quality score more than 30 assigned by Imagen Master 5.0 software
