# Supplementary material for: Identification of seed proteins associated with resistance to pre-harvested aflatoxin contamination in peanut (Arachis hypogaea L)
Source: BMC Plant Biol. 2010 Nov 30;10:267. doi: 10.1186/1471-2229-10-267 (PMC3095339; doi:10.1186/1471-2229-10-267)
Supplement: Additional file 2 — Summary of differential expression of proteins in Yueyou7 and YJ-1 in three treatments. [file 1471-2229-10-267-S2.DOC]

|  | **Yuueyou7** | | **YJ-1** | |
| --- | --- | --- | --- | --- |
|  | **Well-watered condition** | **Drought stress** | **Well-watered condition** | **Drought stress** |
| **Drought stress** | **A: 6**  **B: 9**  **C: 5**  **D: 3** |  | **A: 11**  **B: 12**  **C: 6**  **D: 6** |  |
| ***A. flavus* inoculation under drought stress** | **A: 12**  **B: 11**  **C: 8**  **D: 4** | **A: 8**  **B: 5**  **C: 3**  **C: 2** | **A: 17**  **B: 15**  **C: 5**  **D: 7** | **A: 13**  **B: 8**  **C: 4**  **D: 3** |

A: No. of unique express spot; B: No. of up regulated spot: C: No. of down regulated spot: D: No. of miss spot
